# Pharmacogenetics in Model-Based Optimization of Bevacizumab Therapy for Metastatic Colorectal Cancer

**DOI:** 10.3390/ijms21113753

**Published:** 2020-05-26

**Authors:** Apostolos Papachristos, Eleni Karatza, Haralabos Kalofonos, Gregory Sivolapenko

**Affiliations:** 1 Laboratory of Pharmacokinetics, Department of Pharmacy, School of Health Sciences, University of Patras, 26504 Patra, Greece; alkispapachristos@gmail.com; 2 Department of Pharmacy, School of Health Sciences, National and Kapodistrian University of Athens, 15771 Athens, Greece; ekaratza@pharm.uoa.gr; 3 Institute of Applied and Computational Mathematics (IACM)/Foundation of Research and Technology Hellas (FORTH), 70013 Heraklion, Greece; 4 Division of Medical Oncology, University Hospital of Patras, 26504 Patra, Greece; hkalofon@yahoo.gr

**Keywords:** pharmacogenetics, pharmacokinetics, pharmacodynamics, modeling, bevacizumab

## Abstract

Vascular endothelial growth factor A (VEGF-A) and intercellular adhesion molecule 1 (ICAM-1) are significant regulators of angiogenesis, an important biological process involved in carcinogenesis. Bevacizumab, an anti-VEGF monoclonal antibody (MAB), is approved for the treatment of metastatic Colorectal cancer (mCRC), however clinical outcomes are highly variable. In the present study, we developed a pharmacokinetic (PK), a simplified quasi-steady state (QSS) and a pharmacokinetic/pharmacodynamic (PK/PD) model to identify potential sources of variability. A total of 46 mCRC patients, who received bevacizumab in combination with chemotherapy were studied. *VEGF-A* (rs2010963, rs1570360, rs699947) and *ICAM-1* (rs5498, rs1799969) genes’ polymorphisms, age, gender, weight, and dosing scheme were investigated as possible co-variates of the model’s parameters. Polymorphisms, trough, and peak levels of bevacizumab, and free VEGF-A were determined in whole blood and serum. Data were analyzed using nonlinear mixed-effects modeling. The two-compartment PK model showed that clearance (CL) was significantly lower in patients with mutant *ICAM-1* rs1799969 (*p* < 0.0001), inter-compartmental clearance (Q) was significantly higher with mutant *VEGF-A* rs1570360 (*p* < 0.0001), and lower in patients with mutant *VEGF-A* rs699947 (*p* < 0.0001). The binding QSS model also showed that mutant *ICAM-1* rs1799969 was associated with a lower CL (*p* = 0.0177). Mutant *VEGF-A* rs699947 was associated with a lower free VEGF-A levels, prior to the next dose (*p* = 0.000445). The above results were confirmed by the PK/PD model. Findings of the present study indicated that variants of the genes regulating angiogenesis might affect PK and PD characteristics of bevacizumab, possibly influencing the clinical outcomes.

## 1. Introduction

Angiogenesis is the biological process of new blood vessels formation, an essential process in growth and development, as well as in wound healing. It is also a critical step in the development of cancer from a benign state to a malignant one. This has led to the development and use of angiogenesis inhibitors in the treatment of cancer [1,2]. The most important regulators of angiogenesis in cancer are vascular endothelial growth factors (VEGFs) [3]. The VEGF family consists of six dimeric glycoproteins, which bind to transmembrane receptor tyrosine kinases involved in vascular endothelial cell division [4]. VEGF-A is the most important factor in tumor angiogenesis [5,6]. In addition, several single nucleotide polymorphisms (SNPs) of *VEGF-A* gene are encountered in mammals, such as rs699947, rs1570360, and rs2010963, which result in a different expression of the VEGF glycoprotein. Another significant factor related to angiogenesis was found to be ICAM-1 (Intercellular Adhesion Molecule 1), also known as CD54 (Cluster of Differentiation 54), a protein known to be encoded by the *ICAM-1* gene. ICAM-1 is necessary for leucocyte adherence to capillary endothelium and is an important mediator of tumor migration and invasion [7,8].

Since angiogenesis exerts a vital role in the neoplastic formation, there are several strategies to inhibit this route [9]. These approaches include small molecules, such as tyrosine kinase inhibitors (e.g., sorafenib, sunitinib, pazopanib), and monoclonal antibodies targeting VEGF [9,10,11,12]. Anti-angiogenic therapies lead to tumor stasis, namely to decrease the rate of cell proliferation without inducing apoptosis, thus they are used in combination with other chemotherapeutic drugs [13,14]. 

Bevacizumab was the first anti-VEGF monoclonal antibody (MAB) approved for the treatment of metastatic ColoRectal cancer (mCRC), in combination with chemotherapy [10,15], which led to an increased overall survival in patients with mCRC [16]. Although the default position in the management of mCRC is to add bevacizumab to the chemotherapy backbone, benefits from bevacizumab are modest and clinical outcomes are highly variable [17], with some patients responding remarkably well, while others not.

Several published mathematical models describe the angiogenesis procedures and the anti-angiogenic therapies [18,19,20,21,22]. In this context, the description of the time courses of bevacizumab’s transit through the body and its effect is of paramount importance. However, the pharmacokinetics (PKs) and pharmacodynamics (PDs) of MABs are more complex, compared to typical small molecules. A phenomenon in the disposition of MABs, known as target-mediated drug disposition (TMDD), is the underlying reason why their distribution can be highly influenced by the high-affinity binding to their molecular targets [23,24,25]. This characteristic implies that binding to VEGF-A represents a dominant attribute in bevacizumab’s kinetics. Indeed, at higher bevacizumab concentrations, the TMDD pathway is saturated, and the kinetics appears to be linear. However, when the bevacizumab levels are low, the TMDD clearance pathway dominates, and non-linear kinetics become evident [23]. In recent years, population PK and PD (PK/PD) modeling approaches have been extensively used to describe drug kinetics and effects, to explain between-subject variability and to define individualized dosage regimens, thus allowing the determination of the most desired benefit/risk ratio [26,27,28]. In this vein, several studies have appeared in the literature, trying to elucidate bevacizumab kinetics in humans and other animal species [29,30,31,32,33]. 

Identification of individual variables with a significant impact on pharmacokinetic or pharmacodynamic processes, and their inclusion in adequate models, can lead to enhanced efficacy and safety of the therapeutic agents selected. Co-variate-based optimized therapy has brought significant advantages for cancer patients receiving anticancer treatment [34].

In this study, we have attempted to identify and have found potential predictive markers. To achieve this, we developed three models, namely a PK, a simplified quasi-steady state (QSS) TMDD, and a PK/PD model, utilizing data from patients with mCRC treated with bevacizumab, in combination with oxaliplatin/fluoropyrimidines or irinotecan/fluoropyrimidines chemotherapy. *VEGF-A* (rs2010963, rs1570360, rs699947) and *ICAM-1* (rs5498, rs1799969) genes’ SNPs, age, gender, weight, and dosing scheme were investigated as possible co-variates on the models’ parameters, in an effort to identify potential predictive markers.

## 2. Results

### 2.1. Data

Data analyzed included 156 bevacizumab and 169 free VEGF-A (unbound to bevacizumab) serum concentrations from 46 adult patients with mCRC (Supplementary Materials, Figure S1). No concentration below the limit of quantification was found. The population studied consisted mainly of male patients (61%). The median (IQR) values for weight and age in the population were 74.5 kg (64.15–81.75 kg) and 63 years (53–72 years), respectively. Regarding *VEGF-A* SNPs, 48% of the population had wild-type rs699947, 67% had wild-type rs1570360, and 59% had wild-type rs2010963. Regarding *ICAM-1* SNPs, 30% had wild-type rs5498 and 80% had wild-type 1799969. In terms of the bevacizumab administration, it was predominantly administrated at dose 5 mg/kg, every 2 weeks (76%), and in combination with irinotecan-based chemotherapy (52%).

### 2.2. PK Model 

A two-compartment pharmacokinetic model for intravenous infusion of bevacizumab with first-order elimination was developed. Clearance (CL) and intercompartmental clearance (Q) of bevacizumab were estimated at 0.200 L/day and 0.35 L/day, respectively, while volumes of distribution were estimated at 3.09L in the central (V1) and 2.39L in the peripheral (V2) compartment. A statistically significant correlation was identified between random effects of clearance and intercompartmental clearance (–0.999). The reliability of the estimates might be proved by the low %RSE values (Table 1). The small number of parameters of the pharmacokinetic model with available data allowed for an efficient identification of statistically significant co-variates, with an impact on bevacizumab pharmacokinetics. 

**Figure 1:**
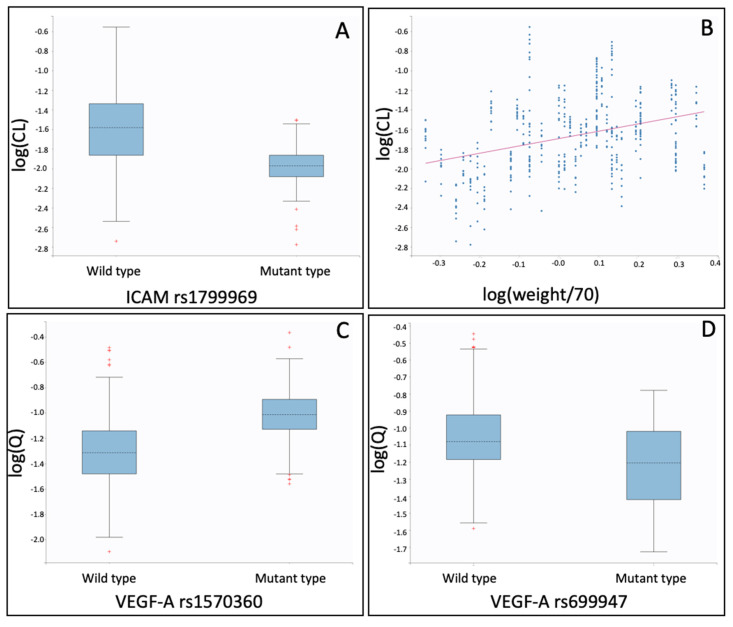
Individual pharmacokinetic parameter estimates of the pharmacokinetic model vs. co-variates identified: (**A**) log(CL: Clearance) vs. *ICAM-1* rs1799969; (**B**) log(CL: Clearance) vs. log(weight/70); and (**C**) log(Q: Intercompartmental clearance) vs. *VEGF-A* rs1570360; (**D**) log(Q: Intercompartmental clearance) vs. *VEGF-A* rs699947.

In the present study, it was found that clearance (CL) of bevacizumab was lower in patients with mutant type *ICAM-1* rs1799969 (Table 1, Figure 1A) and increased with weight (Table 1, Figure 1B). 

It was estimated that the clearance of bevacizumab was given by the following equation:

CL = Clpop * (weight/70)^1.04^ ⋅ exp(−0.423)^cat^ ⋅ exp (hetaCL)

where cat takes value 1 for the mutant *ICAM-1* rs1799969 gene or 0 for the wild-type, CLpop stands for the population parameter estimate of clearance, and hetaCL ~ N(0, ω_CL_^2^) for the random effect of the parameter.

Since clearance seemed to be strongly correlated with the *ICAM-1* rs1799969 polymorphisms, we investigated the differences in the trough bevacizumab levels between the carriers of the mutant and wild-type. Trough levels were significantly higher in carriers of the mutant type (99.1 vs. 57.2 mg/L, *p* = 0.00004) (Supplementary Materials, Figure S2).

Inter-compartmental clearance (Q) was found to be higher in patients with mutant *VEGF-A* rs1570360 (Table 1, Figure 1C) and to be lower in patients with mutant *VEGF-A* rs699947 (Table 1, Figure 1D). 

It was estimated that inter-compartmental clearance of bevacizumab was given by the following equation:

Q = Qpop * exp (0.378)^cat1^ ⋅ exp(−0.429)^cat2^ ⋅ exp (hetaQ)

where cat1 takes the value 1 for the mutant *VEGF-A* rs1570360 gene or 0 for the wild-type, and cat2 takes the value 1 for the mutant *VEGF-A* rs699947 gene, or 0 for the wild-type. Qpop stands for the population parameter estimate of intercompartmental clearance and hetaQ ~ N(0, ω_Q_^2^) for the random effect of the parameter.

The other SNPs studied, as well as the age, gender, co-treatment, and dosing scheme, did not have a statistically significant impact on any model parameter. 

As noted in the relevant visual predictive checks (VPC) (Supplementary Materials, Figure S3), the pharmacokinetic model developed for bevacizumab adequately described the observed data (Supplementary Materials, Figure S4). The distribution and clearance of the antibody, as well as the variability, observed among the study population, were effectively modeled. Moreover, the VPC indicated the good predictive performance of the model developed.

### 2.3. Binding QSS Model 

A binding QSS model was developed and used in order to enhance the understanding of the interaction between the target and the antibody, through their steady-state and elimination constants [35]. Clearance (CL) and intercompartmental clearance (Q) of bevacizumab were estimated to be 0.344 L/day and 0.136 L/day, respectively, while the volumes of distribution of bevacizumab were estimated to be 5.83L in the central (V1) and 3.17L in the peripheral (V2) compartment (Table 2). The information in the present data were not enough to allow for separate estimation of the elimination clearance of the bevacizumab–VEGF-A complex, which was set equal to the CL of free bevacizumab. Baseline VEGF-A (free VEGF-A at time 0, BM0) was estimated to be 0.0137nM, corresponding to the 616.5 ng/L (assuming a 1:1 molecular interaction). The first-order elimination rate constant of free VEGF-A (*k_out_*) was 0.116 day^-1^; and the zero-order production rate constant of free VEGF-A (*k_in_*), which is defined as the typical value of BM_0_ times the typical value of *k_out_* (BM_0,P_ × *k_out,P_*), was 71.51 ng/L/day. The steady-state constant *K*ss, which correlates to the in vivo affinity of bevacizumab to VEGF-A, was found to be 135 nM (Table 2). The QSS constant K_ss_ =K_D_ +k_int_/k_on_ included the dissociation constant (k_off_ /k_on_), which was a measure of affinity between the drug and the target but also the elimination rate constant of the drug–target complex (k_int_) [35].

A statistically significant correlation was found between the random effects of clearance and intercompartmental clearance (−0.999). Parameter estimates presented low %RSE values, indicating that their estimation was accurate in the present population (Table 2).

**Figure 2:**
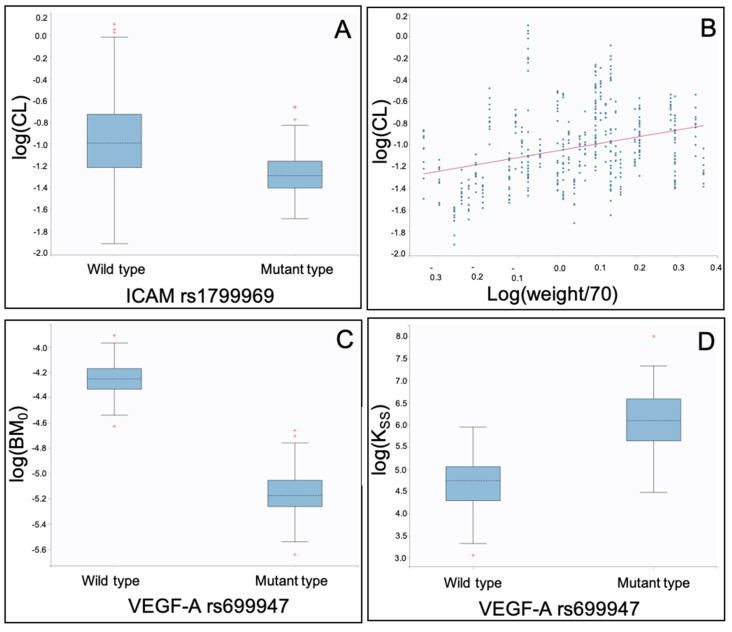
Individual pharmacokinetic parameter estimates of the binding QSS model vs. co-variates identified: (**A**) log(CL: Clearance) vs. *ICAM-1* rs1799969; (**B**) log(CL: Clearance) vs. log(weight/70); (**C**) log(BM_0_: Baseline VEGF-A levels) vs. *VEGF-A* rs699947; and (**D**) log(K_ss_: Steady-state constant) vs. *VEGF-A* rs699947.

As shown in Table 2 and Figure 2, the clearance (CL) of bevacizumab was found to be lower in patients with mutant *ICAM-1* rs1799969 (Table 2, Figure 2A), while it increased with weight (Table 2, Figure 2B). 

The clearance of bevacizumab for each patient might be estimated using the following equation:

CL = Clpop * (weight/70)^1.01^ ⋅ exp(−0.33)^cat^ ⋅ exp (hetaCL)

where cat takes value 1 for the mutant *ICAM-1* rs1799969 gene or 0 for the wild-type. CLpop stands for the population parameter estimate of clearance and hetaCL ~ N(0, ω_CL_^2^) for the random effect of the parameter.

Additionally, it was found that patients with mutant *VEGF-A* rs699947 presented lower baseline free VEGF-A levels (BM0, Table 2, Figure 2C). Moreover, *VEGF-A* rs699947 was found to play a significant role in the binding of bevacizumab with VEGF-A, as patients with the mutant type were found to have a higher steady-state constant *K*ss, potentially indicating a higher in vivo affinity (Table 2, Figure 2D).

The steady-state constant and the baseline free VEGF-A levels for each patient might be estimated using the following equations:

Kss = Kss_pop_ ⋅ exp (1.22)^cat^

BM_0_ = BM_0pop_ ⋅ exp(−0.851)^cat^ ⋅ exp(hetaBM_0_)

where cat takes the value 1 for mutant *VEGF-A* rs699947 gene or 0 for the wild-type. BM_0pop_ stands for the population parameter estimate of baseline VEGF-A levels and hetaBM_0_ ~ N(0, ω_BM0_^2^) is the random effect of the parameter. Kss_pop_ stands for the population parameter estimate of the steady-state constant.

The other SNPs studied, as well as the age, gender, co-treatment, and dosing scheme, were not found to have a statistically significant impact on any model parameter. 

The model adequately described the time course of bevacizumab and free VEGF-A serum concentrations in the study population, as shown by the VPC constructed using the binding model for bevacizumab (Supplementary Materials, Figure S5A) and the free VEGF-A (Supplementary Materials, Figure S5B). The good predicted performance of the model was also evident in the plot (Supplementary Materials, Figure S6).

### 2.4. PK/PD Model

A two-compartment pharmacokinetic model with first-order elimination, coupled with an immediate response Imax model without sigmoidicity, with constant baseline VEGF-A levels was found to most appropriately describe the data (Supplementary Materials, Figure S7). The parameter estimates found were physiologically relevant. Regarding the pharmacokinetic parameter estimates, clearance (CL) and intercompartmental clearance (Q) of bevacizumab were estimated to be 0.388 L/day and 0.315 L/day, respectively; while volumes of distribution were estimated to be 5.48 L in the central (V1) and 8.81 L in the peripheral (V2) compartment. As for the pharmacodynamic part, the maximal inhibitory effect induced by bevacizumab (Imax) was estimated to be 0.951. The concentration of bevacizumab producing 50% VEGF inhibition (IC_50_) was found to be 29.1 mg/L, while the baseline VEGF-A (S_0_) derived was 684 ng/L (Table 3). Random effects of clearance and intercompartmental clearance (–0.979) were found to be significantly correlated. 

**Figure 3:**
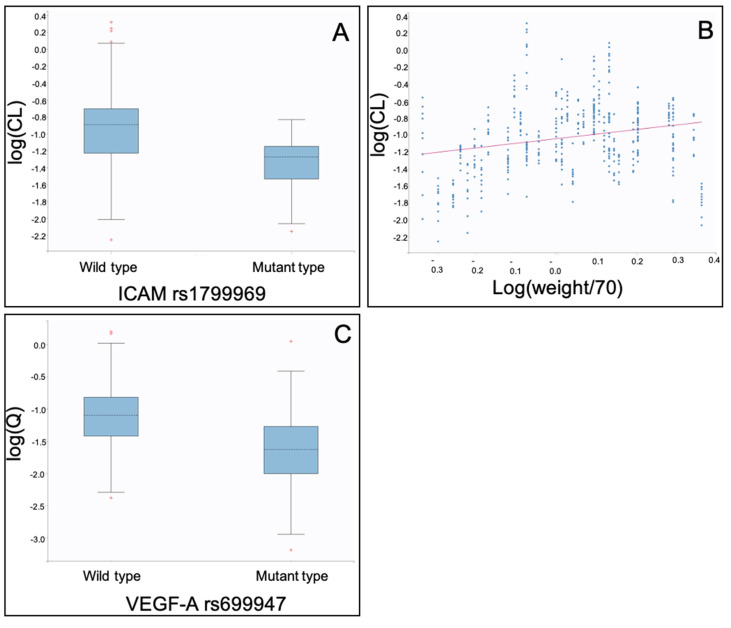
Individual pharmacokinetic parameter estimates of the binding model vs. co-variates identified: (**A**) log(CL: Clearance) vs. *ICAM-1* rs1799969; (**B**) log(CL: Clearance) vs. log(weight/70); and (**C**) log(Q: Intercompartmental clearance) vs. *VEGF-A* rs699947.

As also found with the other two models described herein, the clearance (CL) of bevacizumab was lower in patients with mutant *ICAM-1* rs1799969 (Table 3, Figure 3A), while it increased with weight (Table 3, Figure 3B). 

Clearance of bevacizumab for each patient when using this model was estimated using the following equation:

CL = Clpop * (weight/70)^0.78^ ⋅ exp(−0.423)^cat^ ⋅ exp(hetaCL)

where cat takes the value 1 for the mutant *ICAM-1* rs1799969 gene or 0 for the wild-type. CLpop stands for the population parameter estimate of clearance and hetaCL ~ N(0, ω_CL_^2^) for the random effect of the parameter.

Inter-compartmental clearance (Q) was lower in patients with mutant type gene *VEGF-A* rs699947 (Table 3, Figure 3C). 

The intercompartmental clearance within each patient was given by:

Q = Qpop ⋅ exp (0.378)^cat^ ⋅ exp(hetaQ)

where cat takes the value 1 for the mutant *VEGF-A* rs699947 gene or 0 for the wild-type. Qpop stands for the population parameter estimate of intercompartmental clearance and hetaQ ~ N(0, ω_Q_^2^) for the random effect of the parameter.

The other SNPs studied, as well as the age, gender, co-treatment, and dosing scheme, were not identified as statistically significant co-variates for any parameter.

As shown in the VPC diagrams constructed for bevacizumab (Supplementary Materials, Figure S8A) and free VEGF-A levels (Supplementary Materials, Figure S8B) using the joint PK/PD model, the observed data were adequately described (Supplementary Materials, Figure S9). Inhibition of VEGF-A was well-described in relation to the bevacizumab levels. The diagram also proved to have a good predictive performance of the model developed. 

## 3. Discussion

Given the need for bevacizumab-based treatment optimization in mCRC, in the present study, three models were successfully developed in order to characterize the pharmacokinetic and pharmacodynamic properties of bevacizumab, in patients with mCRC. More specifically, a pharmacokinetic model of bevacizumab, a binding QSS model describing the interaction between bevacizumab and its molecular target VEGF-A, and a pharmacokinetic/pharmacodynamic model describing the inhibition of VEGF-A in relation to the bevacizumab levels were constructed and internally validated. A wide variety of co-variates were investigated for their effect on population parameters of all three models developed, including genetic variants of the *VEGF-A* and *ICAM-1* genes, age, gender, weight, co-treatment, and dosing scheme (Table 4). The aforementioned models described the data satisfactorily and presented a good predictive capacity, while the physiologically relevant parameter estimates were retrieved.

To our knowledge, this was the first PK/PD model developed for bevacizumab in mCRC patients, and it could be used in conjunction with the TMDD QSS model, in order to identify factors that might affect treatment outcomes. The validity of the results retrieved was confirmed by previous population pharmacokinetic studies, where similar estimates of pharmacokinetic parameters were presented [29,31,32,33].

Our data showed that specific SNPs were strongly associated with bevacizumab’s clearance, inter-compartmental clearance, and binding to its molecular target. These associations might explain the variability in the PK and PD properties of bevacizumab, and potentially the observed inter-patient variability in clinical outcomes. 

More specifically, *VEGF-A* rs699947 significantly affects the pharmacokinetics and the binding of bevacizumab with its target. In the PK and the PK/PD models, it was shown that patients with mutant *VEGF-A* rs699947 have a lower intercompartmental clearance (Q) of bevacizumab (Table 1 and Table 3, Figure 1D and Figure 3C). In the binding QSS model, it was shown that patients with mutant type *VEGF-A* rs699947 have a higher steady-state constant (Kss) (Table 2, Figure 2D), which might indicate a higher affinity of bevacizumab to VEGF-A. Thus, this gene by affecting the in vivo binding process of the ligand with its target, also affected its disposition between the two compartments. 

The PK model also revealed a higher inter-compartmental clearance (Q) in patients with mutant-type *VEGF-A* rs1570360 (Table 1, Figure 1C), though without statistically significant alteration of the K_SS._

Both the binding QSS and the PK/PD model describe the bevacizumab concentration in relation to VEGF-A levels, over time. Through these different approaches, baseline (pre-dosing) VEGF-A levels were found to be 616.5ng/L and 684ng/L, respectively (BM_0_ in Table 2 and E_0_ in Table 3). Moreover, from the QSS model, it was found that the *k*out was 0.116 days^-1^, whilst the *k*in (BM0,*P* × *k*out,*P*)—defined as the zero-order production of VEGF-A—was 71.51 ng/L/day (Table 2).

The pharmacokinetic parameter estimates (V1, V2, and CL) of the QSS and the PK/PD models were similar, providing evidence of the precision in their estimation. Despite their similarities, they might offer different information in order to gain a more-in-depth insight into the mechanism of action of the antibody. The first model described the binding, while the second pharmacological action of the antibody, characterized the bevacizumab’s I_max_ and IC_50_ in the study population.

In all three models, both the gene *ICAM-1* rs1799969 (Table 1, Table 2 and Table 3, Figure 1A, Figure 2A and Figure 3A) and the patient’s weight (Table 1, Table 2, Table 3 and Table 4, Figure 1B, Figure 2B and Figure 3B) were identified as statistically significant co-variates that affect the clearance of bevacizumab. Although the weight is known to affect bevacizumab’s clearance, this was the first time that a polymorphism of *ICAM-1*, namely the mutant rs1799969, was found to affect the bevacizumab’s kinetics. Indeed, according to our findings, patients with mutant type *ICAM-1* rs1799969 seemed to have a slower bevacizumab clearance than those with the wild-type and consequently higher bevacizumab trough levels (Supplementary Materials, Figure S1).

Until now, other studies have not investigated the effect of *VEGF-A* and *ICAM-1* polymorphisms on bevacizumab’s PK and PD characteristics. However, the effect of these polymorphisms on clinical outcomes was demonstrated in various cancer types treated with bevacizumab. The mCRC carriers of mutant *VEGF-A* rs699947 and rs1570360 presented significantly prolonged overall survival (OS) and progression-free survival (PFS), compared to the carriers of the wild-type [36,37]. Similarly, mutation of *VEGF-A* rs699947 and rs1570360 are associated with OS benefit in metastatic breast cancer [38]. Additionally, longer PFS was observed in glioblastoma patients who presented *VEGF-A* rs699947 mutations [39]. Thus, the differences in in vivo affinity of bevacizumab to VEGF-A observed in carriers of mutant *VEGF-A* rs699947 might partly explain the differences in clinical outcomes. 

Other studies that have examined the effect of the *ICAM-1* rs1799969 mutations and serum ICAM-1 levels have associated mutant type *ICAM-1* rs1799969 and low serum levels with a prolonged overall survival [37,40,41,42]. Therefore, the slower bevacizumab clearance found in patients with mutant *ICAM-1* rs1799969 might account for the increased efficacy noted in the literature, supporting the use of the *ICAM-1* gene as a prognostic factor of bevacizumab therapy. 

Overall, in this study, it was noted that major angiogenesis variants affected the pharmacokinetic and pharmacodynamic profile of patients with mCRC. It can be expected that differences in in vivo affinity of bevacizumab to VEGF-A reduced the disposition of bevacizumab in tissues observed in patients with mutant *VEGF-A* rs699947, and a slower clearance of bevacizumab seen in patients with mutant *ICAM-1* rs1799969 affect the clinical outcome in terms of survival. Therefore, it could be supported that *VEGF-A* and *ICAM-1* variants might be significant predictive biomarkers of great clinical importance. However, more studies including a larger number of patients and other bevacizumab indications are needed to verify these findings. Future work based on the present study, might also include simulation studies with the models developed herein, more specifically the binding Qss model and the PK/PD model, allowing for a more in-depth mechanistic understanding of bevacizumab’s binding process. In fact, by exploring the impact of these parameters on the bevacizumab and VEGF-A levels, would allow for an investigation on how changes of the steady-state constant are reflected on the Imax and or the IC_50_ of bevacizumab, i.e., its efficacy.

In conclusion, the combination of genetics and mathematical computations transform targeted therapy to precision medicine. This confirms that the utilization of genetic information leads to the optimization of cancer treatment [43]. Our data demonstrated that identification of genetic variants play a role in a drug’s pharmacokinetics and can have an impact on the safety and efficacy profile of bevacizumab. Population PK/PD analyses, which include genetic data as co-variates, appear to be an important tool for dose optimization. We expect that approaches such as ours will gain the support and the trust of not only the medical community but also of the regulatory authorities and health technology assessors.

## 4. Materials and Methods

### 4.1. Cohort

Data from 46 adult patients with histologically confirmed mCRC (ECOG performance status 0-2) were analyzed. All patients were treated as per the standard clinical practice at the Department of Oncology, University Hospital of Patras, Patra, Greece. The patients received bevacizumab (Avastin^®^, Roche, Basel, Switzerland) in combination with oxaliplatin-or irinotecan-and fluoropyrimidine-based chemotherapy. Bevacizumab was administered as an intravenous infusion at a dose of 5 mg/kg, once every 2 weeks, in combination with 5-fluorouracil/leucovorin/irinotecan or oxaliplatin (BEV-FOLFIRI or BEV-FOLFOX, respectively) or at a dose of 7.5 mg/kg, once every 3 weeks, in combination with capecitabine/irinotecan or oxaliplatin (BEV-CapIRI or BEV-CapOX, respectively), every 3 weeks. The study was conducted in accordance with the International Conference of Harmonisation (ICH) Good Clinical Practice. Approval was obtained by the Hospital’s Ethics Committee. Prior to enrolment, all patients provided signed informed consent.

### 4.2. Samples

Whole blood samples were collected on cycle 1 and several cycles during the treatment for measurement of trough and peak levels of bevacizumab and free VEGF-A. In addition, an extra sample was collected for the detection of *VEGF-A* and *ICAM-1* SNPs. In total 169 samples (87 pre-dose and 82 post-dose) were collected and analyzed for free VEGF-A and 156 samples (76 pre-dose and 80 post-dose) for bevacizumab. Blood samples were collected in serum separator tubes and allowed to clot for 30 min. After centrifugation at 1000×g for 20 min, the serum was removed and stored in aliquots at ≤−20 °C until analysis.

### 4.3. Genotyping

Genomic DNA was extracted from the peripheral blood leucocytes from patients, using the Gentra Puregene Blood kit (QIAGEN). DNA concentrations were determined by measuring the optical density at 260 nm with a UV–Vis spectrophotometer (NanoDrop 2000, Thermo Fisher Scientific, Waltham, MA, USA). DNA purity, which is indicated by the ratio of optical density at 260 and 280 nm, was 1.7–1.9. Genomic variants were analyzed by polymerase chain reaction (PCR), according to the KAPA2G Fast HotStart protocol (KAPABIOSYSTEMS, Wilmington, MA, USA); detailed information per genomic variant amplification conditions is available upon request.

### 4.4. Measurement of Bevacizumab Levels

The bevacizumab levels were measured in patients’ serum, utilizing a validated enzyme-linked immunosorbent assay (ELISA), as previously described [33,44]. This method measured bevacizumab carrying one molecule of or no VEGF, with a detection limit of 0.033 mg/L, linearity range 5–75 mg/L, and intraday CV% 5.6 % [44]. In brief, as per literature, positive control of 0.24, 0.47, 0.94, 1.88, 3.75, 7.5, 15, and 30 mg/L of bevacizumab (Avastin^®^, Roche) was prepared for the standard curve. Test samples were diluted 1:100 in 1% PBS–BSA. Both, the test samples and the standards were prepared in duplicates. The optical density was measured at 450 nm, with a correction at 650 nm, using an ELISA plate reader (ThermoMax, Molecular Devices, San Diego, CA, USA). Data were linearized by plotting the log of concentrations versus the optical density and the best-fit line of the standard curve was determined by regression analysis (OriginPro 8.0 software, OriginLab^®^ Corporation, Northampton, MA, USA). Finally, the test sample concentrations found were corrected by the dilution factor [33,44].

### 4.5. Measurement of Free VEGF-A

Free VEGF levels were measured by a commercially available ELISA kit (Quantikine^®^ Human VEGF, R&D Systems^®^, Minneapolis, MN, USA), which detects VEGF-A in human serum, with a detection limit of 9 ng/L, a linearity range of 82%–107%, and an intra-assay CV% 6.7% [45]. As per the manufacturer’s instructions, the standard curve was generated with VEGF concentrations ranging from 31.2 to 2000 ng/L. All standards and samples were prepared in duplicates and the optical density was measured at 450 nm, with a correction at 550 nm, using an ELISA plate reader (ThermoMax, Molecular Devices). Data were linearized by plotting the log of the concentrations versus the log of the optical density, and the best-fit line of the standard curve was determined by regression analysis (OriginPro 8.0 software, OriginLab^®^ Corporation, Northampton, MA, USA) [45].

### 4.6. Model Development and Co-Variate Assessment 

Data were analyzed using nonlinear mixed-effects modeling. The software utilized was the Monolix^®^ Suite 2018R2 (Lixoft, Orsay France). Population parameters estimation was performed using the Stochastic Approximation Expectation–Maximization (SAEM) algorithm, while the objective function value was estimated using the Importance Sampling Monte Carlo at the final population values. All parameters were assumed to follow a lognormal distribution, except for the parameter representing the maximal drug-induced inhibition (Imax) of VEGF-A, which was assumed to follow a logit-normal distribution, ranging from 0 to 1. An exponential model was used to describe between-subject variability (BSV), ensuring positive values. The random effect heta (η) of each parameter followed a normal distribution with a mean of zero and an estimated variance of ω^2^. The %RSE, i.e., the relative standard error of the model parameters, was obtained by the Fisher Information Matrix, computed using stochastic approximation.

Initial estimates used in the present modeling exercise were the parameter estimates reported in Panoilia et al., for the PK and the TMDD model. For the PK part of the PK/PD model, initial estimates used were those estimated using the PK model developed in the present study. Initial estimates used for CL (clearance of bevacizumab) and V1 (volume of distribution in the central compartment) were used as priors (Bayesian estimation) in the TMDD model and the PK part of the PK/PD model.

After structural model building, various residual error models, i.e., constant, proportional and combined were explored using the likelihood ratio test.

Continuous co-variate weight (centered around 70 kg) and age (centered on median) were tested, both linearly and allometrically. Categorical co-variates, namely gender, dosing scheme, co-treatment, *VEGF-A* and *ICAM-1* SNPs were assumed to affect the system linearly. Independence between gender and SNPs, as well as between the different *ICAM-1* and *VEGF-A* SNPs was ensured, using the chi-square test of independence with and without Yate’s continuity correction, as well as Fisher’s exact test, given the sample size (Supplementary Materials, Tables S1–S3). Statistical tests were performed using validated R (R Core Team, 2018, Vienna, Austria) functions. No statistically significant correlation was found with an alpha level of 0.05.

The effect of co-variates was investigated both by univariate analysis and by a combination of a stepwise forward addition and backward elimination process. A significance level of 5% was considered in both procedures. Statistical significance of the co-variates was initially assessed using a Pearson correlation test for continuous co-variates and a one-way ANOVA for categorical co-variates. Then, a Wald test and a likelihood ratio test for both continuous and categorical co-variates were used, in order to decide the inclusion in the model. The precision of the co-variate’s parameter estimate and goodness of fit statistical criteria were also considered.

Correlation between random effects was also investigated, by estimation of the Pearson correlation coefficient and Pearson correlation test. Statistically significant correlations (*p*-value < 0.05) resulting in high (> 0.5) population coefficients of correlation and low (< 35%) %RSE, were included in the model.

### 4.7. PK Model

Based on previous studies, where a two-compartment model with first-order elimination was found appropriate to describe bevacizumab pharmacokinetics, a similar model was developed [29,30,31,32,33]. In view of its simplicity, encompassing a rather small number of parameters in relation to data available, assessment of the co-variates’ effect was made possible.

### 4.8. Binding QSS Model

Using a simplification of the general target-mediated drug distribution model [23] assuming quasi-steady state (QSS) [33,35]; a QSS model was constructed, which was able to simultaneously describe the binding process of bevacizumab with its target VEGF-A, bevacizumab’s pharmacokinetics, and free VEGF-A levels, i.e., concentration of VEGF-A that was unbound to bevacizumab [33]. This previously developed model was used in the present analysis. Concentrations were expressed in nM for simultaneous modeling of VEGF-A and the bevacizumab’s levels. The molecular weight of bevacizumab and VEGF-A used were 149kDa and 45kDa, respectively [46].

### 4.9. PK/PD Model

According to Avastin SmPC, the mechanism of action of bevacizumab relies on the neutralization of the biological activity of VEGF-A [47]. Indeed, the antibody binds to its target (VEGF-A) and prevents it from vascularizing tumors, inhibiting tumor growth. Therefore, free VEGF-A (unbound to bevacizumab) levels might be indicative of the pharmacodynamic action of the compound [48]. In order to simultaneously characterize the pharmacokinetics and pharmacodynamics of bevacizumab, a joint PK/PD model was developed. Thus, the inhibition of free VEGF-A levels (ng/L) was modeled in conjunction with bevacizumab levels (mg/L). Given that the PK structural model is known, a variety of structural pharmacodynamic models was explored. Immediate response Imax models, with and without sigmoidicity, with a constant, linear, and exponential decrease of baseline free VEGF-A levels were tested. All parameters were considered to follow a lognormal distribution, except for Imax that follows a logit-normal distribution, ranging from 0 to 1. Models were compared in terms of −2*log likelihood (–2LL) for nested models’ confrontation, Akaike and Bayesian information criteria (AIC and BIC, respectively), for selection between nonhierarchical models. A reduction of statistical criteria greater than 3.84 was designated as statistically significant at *p* < 0.05. A simultaneous approach for parameter estimation of both the pharmacokinetic and pharmacodynamic parameters was chosen, as it is known to be more reliable [42].

### 4.10. Model Evaluation

The precision of parameter estimates, as expressed through relative standard errors (%RSE) was assessed to determine the reliability of the estimations. Various diagnostic goodness of fit plots was considering for the evaluation of the models developed, including population predictions (PRED) overlaid on observations (OBS) versus time or concentration, and normalized prediction distribution errors (NPDE) versus time or concentrations.

The predictive performance of the models was evaluated with prediction-corrected visual predictive checks (VPC) and numerical predictive check plots generated using Monte Carlo simulation of 1000 datasets and 90% prediction intervals.

## Figures and Tables

**Table 1:** Parameter estimates of the pharmacokinetic model.

Fixed Effects (Unit)	Parameter Value	Standard Error	RSE (%)	*p* Value
CLpop(L/day)	0.200	0.0157	7.8	
*ICAM-1* rs1799969 mutant on CL	−0.423	0.0298	7.0	<2.2e–16
log(weight/70) on CL	1.04	0.0701	6.8	<2.2e–16
V1pop (L)	3.09	0.196	6.4	
Qpop (L/day)	0.35	0.0142	4.1	
*VEGF-A* rs1570360 mutant on Q	0.378	0.0167	4.4	<2.2e–16
*VEGF-A* rs699947 mutant on Q	−0.429	0.0192	4.5	<2.2e–16
V2pop (L)	2.39	0.804	33.6	
**Standard Deviation of the Random Effects**	**Parameter Value**	**Standard Error**	**RSE (%)**	
ω_CL_	0.319	0.0648	20.3	
ω_V1_	0.174	0.0487	28.0	
ω_Q_	0.160	0.039	24.4	
ω_V2_	0.676	0.253	37.4	
**Proportional Error Model**	**Parameter Value**	**Standard Error**	**RSE (%)**	
σ_prop_	0.246	0.0198	8.1	
**Correlation**	**Coefficient**	**Standard Error**	**RSE (%)**	
p(Q,CL)	−0.999	0.22	22.0	

RSE relative standard error.. Population parameter estimates of clearance (CLpop), volume of distribution in the central compartment (V1pop), intercompartmental clearance (Qpop), volume of distribution in the peripheral compartment (V2pop), ω standard deviation of random effects representing inter-individual variability for clearance (ω_CL_), volume of distribution in the central compartment (ω_V1_), intercompartmental clearance (ω_Q_), volume of distribution in the peripheral compartment (ω_V2_), σ_prop_ proportional error, and Pearson correlation co-efficient between random effects of clearance and intercompartmental clearance p(Q,CL).

**Table 2:** Parameter estimates of the binding quasi-steady state (QSS) model.

Fixed Effects (Units)	Parameter Value	Standard Error	RSE (%)	*p* Value
V1pop (L)	5.83	0.335	5.7	
K_outpop_ (day ^−1^)	0.116	0.0285	24.6	
K_SSpop_ (nM)	135	46.5	34.4	
*VEGF-A* rs699947 mutant on K_SS_	1.22	0.394	32.3	0.00198
BM_0pop_ (nM) (or ng/L)	0.0137 (616.5)	0.00256	18.8	
*VEGF-A* rs699947 mutant on BM_0_	−0.851	0.242	28.5	0.000445
CL(L/day)	0.344	0.0205	5.9	
*ICAM-1* rs1799969 mutant on CL	−0.33	0.139	42.2	0.0177
log(weight/70) on CL	1.01	0.314	31.1	0.00129
Qpop (L/days)	0.136	0.00795	5.8	
V2pop (L)	3.17	1.23	38.7	
**Standard Deviation of the Random Effects**	**Parameter Value**	**Standard Error**	**RSE(%)**	
ω_V1_	0.169	0.0535	31.6	
ω_ΒΜ0_	0.24	0.0547	22.8	
ω_CL_	0.309	0.046	14.9	
ω_Q_	0.201	0.0721	35.9	
ω_V2_	0.555	0.223	40.2	
**Proportional Error Model**	**Parameter Value**	**Standard Error**	**RSE (%)**	
σ_BEVA_	0.253	0.0186	7.3	
σ_VEGF_	0.290	0.0279	9.6	
**Correlation**	**Coefficient**	**Standard Error**	**RSE (%)**	
p(Q,CL)	-0.999	0.367	36.8	

RSE relative standard error. Population parameter estimate of the central volume of distribution (V1pop), the elimination rate constant of VEGF-A (K_outpop_), the steady-state constant (K_sspop_), the baseline VEGF-A levels (BM_0pop_), the clearance (CLpop), the intercompartmental clearance (Qpop), the peripheral volume of distribution (V2pop), ω standard deviation of random effects representing inter-individual variability for volume of distribution in the central compartment (ω_V1_), baseline VEGF-A levels (ω_ΒΜ0_), clearance (ω_CL_), intercompartmental clearance (ω_Q_), volume of distribution in the peripheral compartment (ω_V2_), σ_BEVA_ proportional error for bevacizumab concentrations, σ_VEGF_ proportional error for VEGF-A concentrations, and Pearson correlation co-efficient between random effects of clearance and intercompartmental clearance p(Q,CL).

**Table 3:** Parameter estimates of the pharmacokinetic/pharmacodynamic (PK/PD) model.

Fixed Effects (Units)	Parameter Value	Standard Error	RSE (%)	*p* Value
CLpop (L/days)	0.388	0.0288	7.4	
*ICAM-1* rs1799969 mutant on CL	−0.423	0.153	36.1	0.00566
log(weight/70) on CL	0.78	0.243	31.2	0.0228
V1pop (L)	5.48	0.28	5.1	
Qpop (L/days)	0.315	0.0362	11.5	
VEGF-A rs699947 mutant on Q	−0.414	0.13	31.4	<2.2e–16
V2(L)	8.81	2.3	26.1	
E_0pop_ (ng/L)	684	105	15.4	
I_maxpop_	0.951	0.0251	2.6	
IC_50pop_ (mg/L)	29.1	7.49	25.7	
**Standard Deviation of the Random Effects**	**Parameter Value**	**Standard Error**	**RSE (%)**	
ω_CL_	0.338	0.0586	17.4	
ω_V1_	0.176	0.0646	36.7	
ω_Q_	0.601	0.173	28.8	
ω_V2_	0.579	0.189	32.6	
ω_E0_	0.167	0.0591	35.4	
**Proportional Error Model**	**Parameter Value**	**Standard Error**	**RSE (%)**	
σ_BEVA_	0.238	0.02	8.4	
σ_VEGF_	0.264	0.0297	11.3	
**Correlation**	**Coefficient**	**Standard Error**	**RSE (%)**	
p(Q,CL)	−0.979	0.0695	7.1	

RSE relative standard error. Population parameter estimates of bevacizumab clearance (CLpop), volume of distribution in the central compartment (V1pop), intercompartmental clearance (Qpop), volume of distribution in the peripheral compartment (V2pop), baseline VEGF-A levels (E0pop ), maximal VEGF-A inhibition effect of bevacizumab (I_maxpop_), bevacizumab concentration producing 50% inhibition of VEGF-A (IC_50pop_), ω standard deviation of random effects representing inter-individual variability for clearance (ωCL), volume of distribution in the central compartment (ωV1), intercompartmental clearance (ωQ), volume of distribution in the peripheral compartment (ωV2), baseline VEGF-A levels (ωE0), σBEVA proportional error for bevacizumab concentrations, σVEGF proportional error for VEGF-A concentrations, and Pearson correlation co-efficient between random effects of clearance and intercompartmental clearance p(Q,CL).

**Table 4:** Significant co-variate effects observed in this study.

Model	Parameter	Co-Variate
**PK model**	CL	*ICAM-1* rs1799969
		Weight
	Q	*VEGF-A* rs1570360
		*VEGF-A* rs699947
**Binding QSS model**	K_ss_	*VEGF-A* rs699947
	BM_0_	*VEGF-A* rs699947
	CL	*ICAM-1* rs1799969
		Weight
**PK/PD model**	CL	*ICAM-1* rs1799969
		Weight
	Q	*VEGF-A* rs699947

## Supplementary Materials

The following are available online at https://www.mdpi.com/1422-0067/21/11/3753/s1, Figure S1: Bevacizumab concentrations in mg/L (A) and free VEGF concentrations in ng/L (B), Figure S2. Bevacizumab trough levels (mg/L) for ICAM-1 rs1799969 wild-type vs. mutant. Carriers of the mutant type presented significantly higher trough levels compared to carriers of the wild-type (p = 0.00004), Figure S3. Prediction-corrected visual predictive checks (VPC) of the pharmacokinetic model developed for bevacizumab using 1000 Monte Carlo simulations. Median (solid line), 10th, and 90th percentiles (blue line) of the observed data overlaid to the 95% confidence intervals (colored areas) for the median, 10th, and 90th percentiles of the simulated data, Figure S4. Observed VS Predicted for the PK model., Figure S5. Prediction-corrected visual predictive checks (VPC) of the binding (QSS) model developed for bevacizumab (A) and VEGF (B) using 1000 Monte Carlo simulations. Median (solid line), 10th, and 90th percentiles (blue line) of the observed data overlaid to the 95 % confidence intervals (colored areas) for the median, 10th, and 90th percentiles of the simulated data., Figure S6. Observed VS Predicted for the TMDD model (right Bevacizumab in nM), (left VEGF in nM)., Figure S7. Schematic representation of the PK/PD model developed in the present analysis. Inf: administration of bevacizumab by infusion, V1: the volume of distribution in the central compartment, CL: the clearance of elimination, Q: the inter-compartmental clearance, V2: the volume of distribution of second compartment, C(t): Concentration of bevacizumab in the central compartment, E0: VEGF levels before bevacizumab administration, E(t): free-VEGF levels that have not been neutralized after bevacizumab administration., Figure S8. Prediction-corrected visual predictive checks (VPC) of the PK/PD model developed for bevacizumab (A) and VEGF (B) using 1000 Monte Carlo simulations. Median (solid line), 10th, and 90th percentiles (blue line) of the observed data overlaid to the 95 % confidence intervals (colored areas) for the median, 10th, and 90th percentiles of the simulated data., Figure S9. Observed VS Predicted for the PK/PD model (right Bevacizumab in mg/mL), (left VEGF in ng/mL). Table S1: Gender vs. SNPs, Table S2. ICAM-1 rs1799969 vs. SNPs, Table S3. ICAM-1 rs5498 vs. SNPs.
