# Supplementary material for: Pharmacogenetics in Model-Based Optimization of Bevacizumab Therapy for Metastatic Colorectal Cancer
Source: Int J Mol Sci. 2020 May 26;21(11):3753. doi: 10.3390/ijms21113753 (PMC7311957; doi:10.3390/ijms21113753)
Supplement: Supplementary file 1 [file ijms-21-03753-s001.pdf]

Supplementary Materials:

Table S1. Gender vs. SNPs.

	chi squared with continuity correction .p.value	chi squared .p.value	Fisher.test p.value
VEGF-A rs699947	0.967	0.967	1
VEGF-A rs1570360	0.4512	0.4512	0.46832563
VEGF-A rs2010963	0.7017	0.7017	0.75664848
ICAM-1 rs5498	0.6744	0.6744	0.73346226
ICAM-1 rs1799969	0.9868	0.6911	1

Table S2. ICAM-1 rs1799969 vs. SNPs.

	chi squared with continuity correction .p.value	chi squared .p.value	Fisher.test p.value
VEGF-A rs699947	0.122	0.122	0.19007478
VEGF-A rs1570360	0.6297	0.6297	0.62288582
VEGF-A rs2010963	0.1129	0.1129	0.19025932
ICAM-1 rs5498	0.0451	0.0451	0.04557559
Sex	0.9868	0.6911	1

Table S3. ICAM-1 rs5498 vs. SNPs.

	chi squared with continuity correction .p.value	chi squared .p.value	Fisher.test p.value
VEGF-A rs699947	0.3568	0.3568	0.39153924
VEGF-A rs1570360	0.149	0.149	0.21304503
VEGF-A rs2010963	0.1711	0.1711	0.17514671
ICAM-1 rs1799969	0.0451	0.0451	0.04557559
Sex	0.6744	0.6744	0.73346226

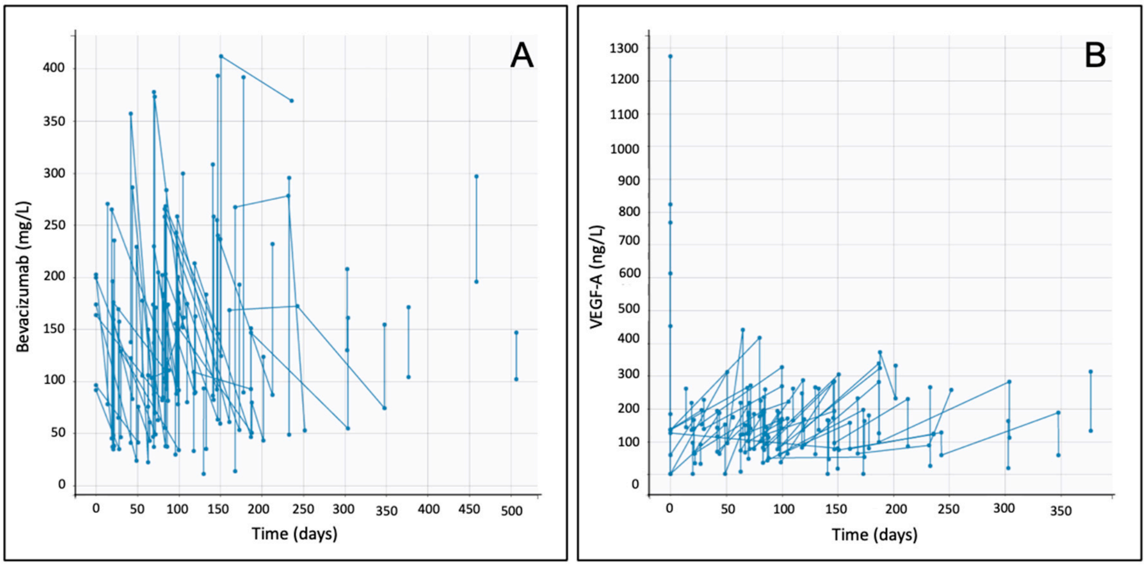

Figure S1. Bevacizumab concentrations in mg/L (A) and free VEGF concentrations in ng/L (B).

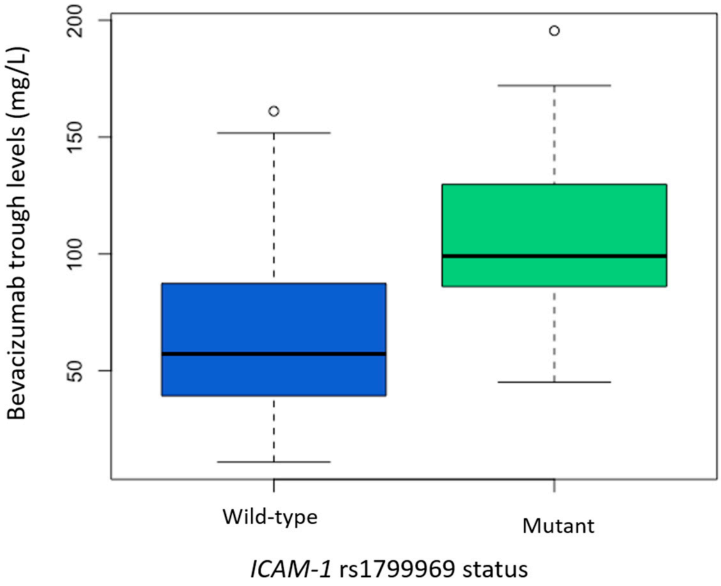

Figure S2. Bevacizumab trough levels (mg/L) for ICAM-1 rs1799969 wild-type vs. mutant. Carriers of the mutant type presented significantly higher trough levels compared to carriers of the wild-type ($p=0.00004$).

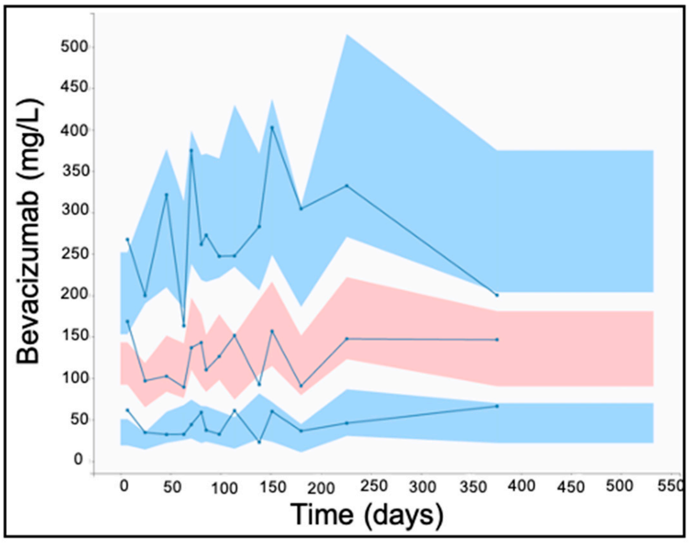

Figure S3. Prediction-corrected visual predictive checks (VPC) of the pharmacokinetic model developed for bevacizumab using 1000 Monte Carlo simulations. Median (solid line), 10th, and 90th percentiles (blue line) of the observed data overlaid to the 95 % confidence intervals (colored areas) for the median, 10th, and 90th percentiles of the simulated data.

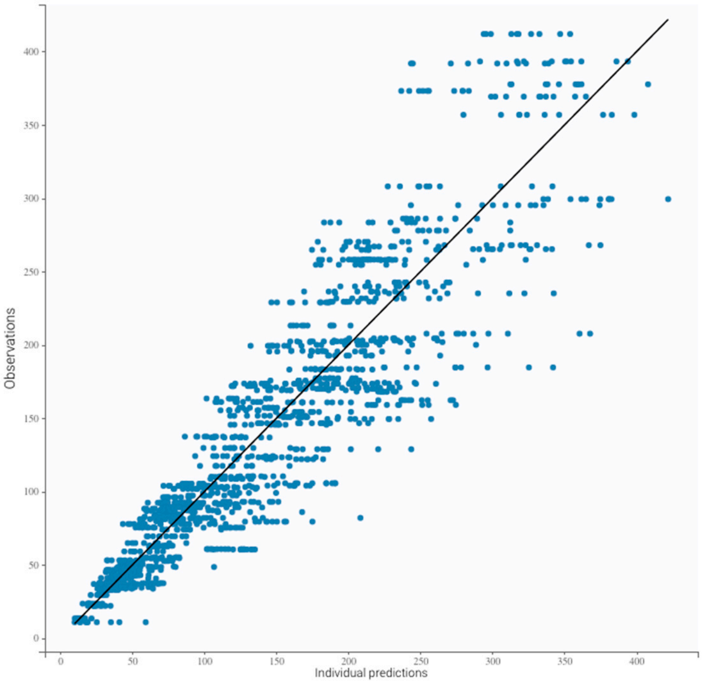

Figure S4. Observed VS Predicted for the PK model.

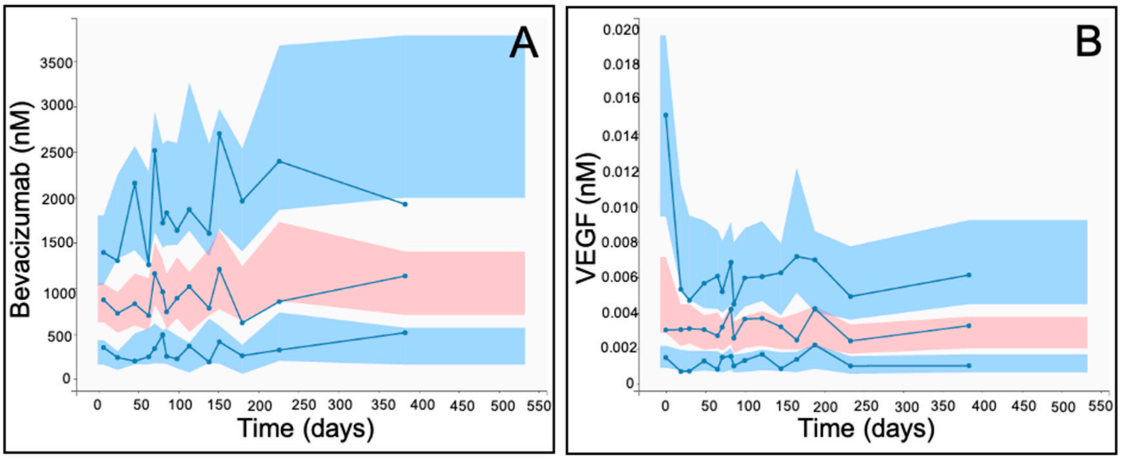

Figure S5. Prediction-corrected visual predictive checks (VPC) of the binding (QSS) model developed for bevacizumab (A) and VEGF (B) using 1000 Monte Carlo simulations. Median (solid line), 10th, and 90th percentiles (blue line) of the observed data overlaid to the 95 % confidence intervals (colored areas) for the median, 10th, and 90th percentiles of the simulated data.

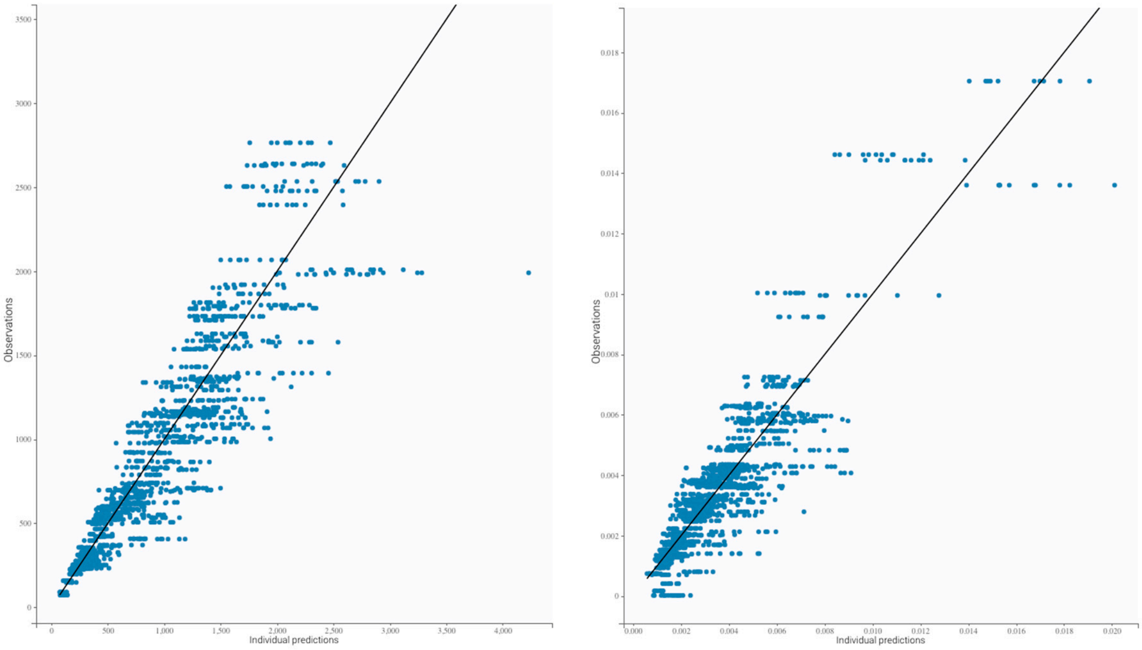

Figure S6. Observed VS Predicted for the TMDD model (right Bevacizumab in nM), (left VEGF in nM).

PK/PD model

Two-compartment pharmacokinetic model of bevacizumab with administration the intravenous route (multiple infusions) and linear elimination, coupled to a direct Imax model of VEGF effect neutralization.

PK part of the model:

$$C(t)=\begin{cases}\sum_{i=1}^{n-1}\frac{D_i}{Tinf_i}\left[\begin{array}{c}\frac{A}{\alpha}\left(1-e^{-\alpha Tinf_i}\right)e^{-\alpha\left(t-t_{D_i}-Tinf_i\right)}\\+\frac{B}{\beta}\left(1-e^{-\beta Tinf_i}\right)e^{-\beta\left(t-t_{D_i}-Tinf_i\right)}\end{array}\right] & \\ \quad+\frac{D}{Tinf_n}\left[\begin{array}{c}\frac{A}{\alpha}\left(1-e^{-\alpha(t-t_{D_n})}\right)\\+\frac{B}{\beta}\left(1-e^{-\beta(t-t_{D_n})}\right)\end{array}\right] & \text{if } t-t_{D_n}\leq Tinf,\\ \sum_{i=1}^{n}\frac{D_i}{Tinf_i}\left[\begin{array}{c}\frac{A}{\alpha}\left(1-e^{-\alpha Tinf_i}\right)e^{-\alpha\left(t-t_{D_i}-Tinf_i\right)}\\+\frac{B}{\beta}\left(1-e^{-\beta Tinf_i}\right)e^{-\beta\left(t-t_{D_i}-Tinf_i\right)}\end{array}\right] & \text{if not.}\end{cases}$$

- $A=\frac{1}{V_1}\frac{\alpha-\frac{Q}{V_2}}{\alpha-\beta}$

- $B=\frac{1}{V_1}\frac{\beta-\frac{Q}{V_2}}{\beta-\alpha}$

- $\alpha=\frac{k_{21}k}{\beta}=\frac{\frac{Q}{V_2}\frac{CL}{V_1}}{\beta}$

- $\beta=\begin{cases}\frac{1}{2}\left[k_{12}+k_{21}+k-\sqrt{(k_{12}+k_{21}+k)^2-4k_{21}k}\right]\\ \frac{1}{2}\left[\frac{Q}{V_1}+\frac{Q}{V_2}+\frac{CL}{V_1}-\sqrt{\left(\frac{Q}{V_1}+\frac{Q}{V_2}+\frac{CL}{V_1}\right)^2-4\frac{Q}{V_2}\frac{CL}{V_1}}\right]\end{cases}$

Parameters of the PK part of the model

V_1: the volume of distribution in the central compartment, k: the elimination rate constant, CL: the clearance of elimination, Q: the inter-compartmental clearance, V_2: the volume of distribution of second compartment, k_{12}: the distribution rate constant from compartment 1 to compartment 2, k_{21},: the distribution rate constant from compartment 2 to compartment 1, n: number of doses, T inf_i: duration of infusion for i multiple doses, D $_i$:is the total i^{th} administered dose for multiple doses. t: time after n doses D_i (i = 1, ...n) given at time t_{D_i} (t ≥ t_{D_n}) For multiple doses, the delay between successive doses is supposed to be constant and to be greater than infusion duration (t_D − t_D = constant and t_D − t_D > T inf_i for infusion).

PD part of the model:

Concentration of bevacizumab in the central compartment C(t) is given by the equations described by the PK model above

$E(t) = E0 * (\_?\, 1 - \frac{Imax\_?*C(t)}{C(t)+IC50}\_?)$

Parameters of the PD part of the model:

E0: VEGF levels before bevacizumab administration, Imax: maximal fraction of inhibition of the VEGF biologic effect by bevacizumab, IC50: half-maximal inhibitory concentration of bevacizumab, E(t): free-VEGF levels that have not been neutralized after bevacizumab administration

Schematic representation of the model

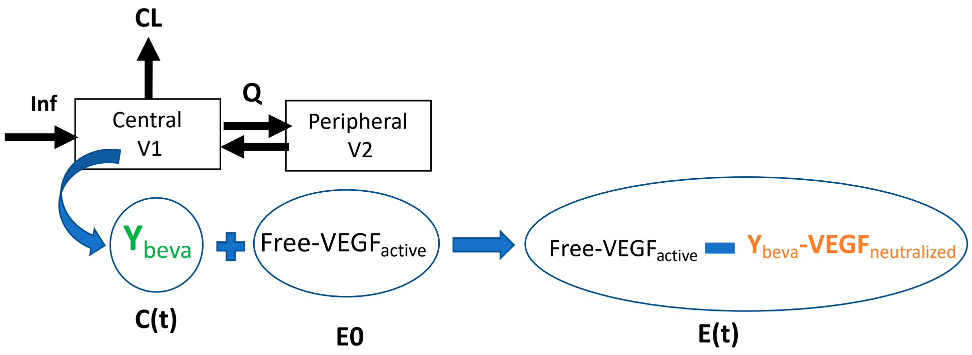

Figure S7. Schematic representation of the PK/PD model developed in the present analysis. Inf: administration of bevacizumab by infusion, V1: the volume of distribution in the central compartment, CL: the clearance of elimination, Q: the inter-compartmental clearance, V2: the volume of distribution of second compartment, C(t): Concentration of bevacizumab in the central compartment, E0: VEGF levels before bevacizumab administration, E(t): free-VEGF levels that have not been neutralized after bevacizumab administration.

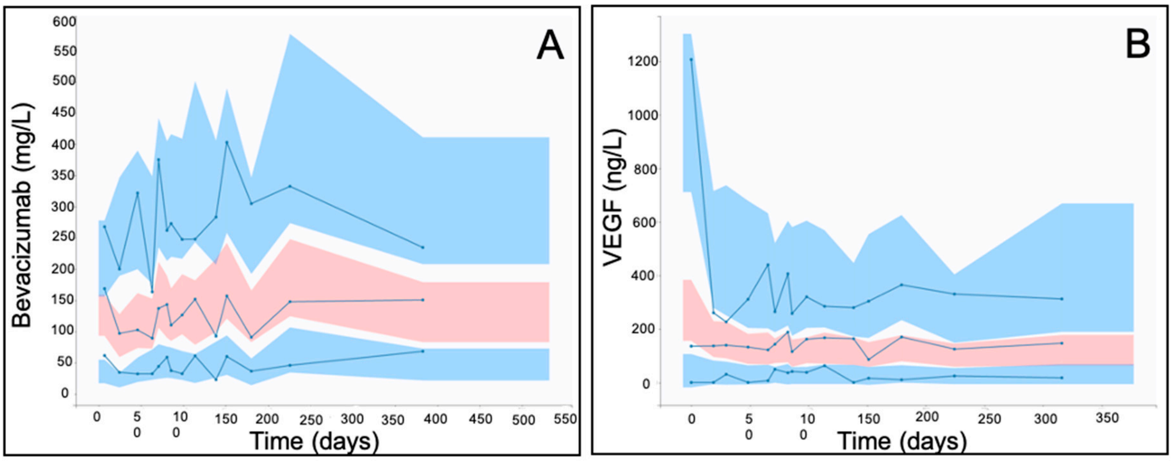

Figure S8. Prediction-corrected visual predictive checks (VPC) of the PK/PD model developed for bevacizumab (A) and VEGF (B) using 1000 Monte Carlo simulations. Median (solid line), 10th, and 90th percentiles (blue line) of the observed data overlaid to the 95 % confidence intervals (colored areas) for the median, 10th, and 90th percentiles of the simulated data.

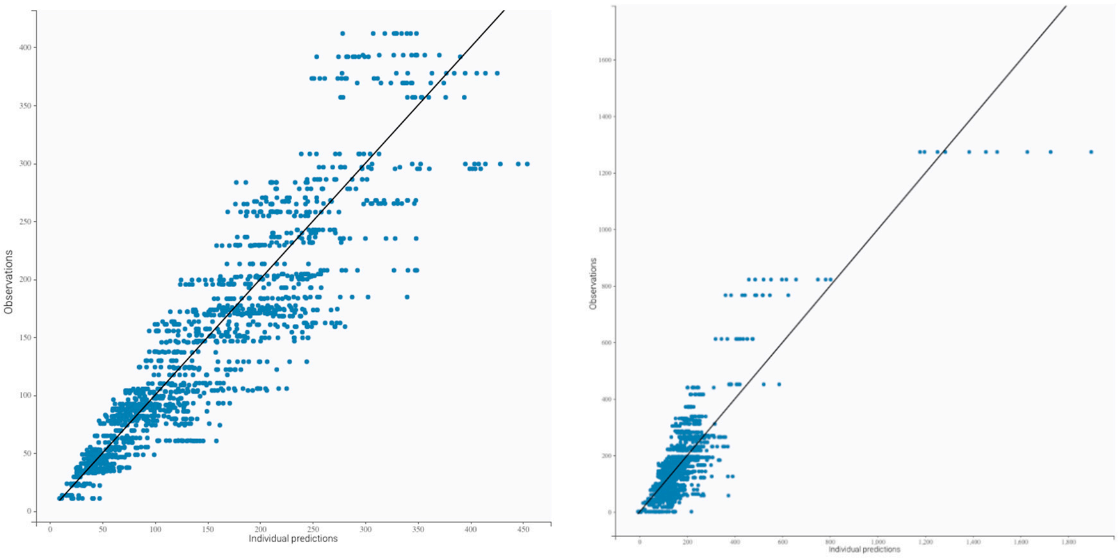

Figure S9. Observed VS Predicted for the PK/PD model (right Bevacizumab in mg/mL), (left VEGF in ng/mL).
